# Increased Participation and Decreased Performance in Recreational Master Athletes in “Berlin Marathon” 1974–2019

**DOI:** 10.3389/fphys.2021.631237

**Published:** 2021-06-28

**Authors:** Marlen Reusser, Caio Victor Sousa, Elias Villiger, José Ramón Alvero Cruz, Lee Hill, Thomas Rosemann, Pantelis T. Nikolaidis, Beat Knechtle

**Affiliations:** ^1^Institute of Primary Care, University of Zurich, Zurich, Switzerland; ^2^Bouvé College of Health Sciences, Northeastern University, Boston, MA, United States; ^3^Dpto de Fisiología Humana, Histología, Anatomia, Patológica y Educación Física y Deportiva Universidad de Málaga, Málaga, Spain; ^4^Division of Gastroenterology & Nutrition, Department of Pediatrics, McMaster University, Hamilton, ON, Canada; ^5^Exercise Physiology Laboratory, Nikaia, Greece; ^6^Medbase St. Gallen Am Vadianplatz, St. Gallen, Switzerland

**Keywords:** marathon, running, participation, performance, age of peak performance, performance decline, sex differences in endurance

## Abstract

The aspect of participation and performance trends in marathon running has been investigated mainly in marathons held in the United States of America (e.g., “New York City Marathon,” “Boston Marathon”), but not for the fastest course in the world, the “Berlin Marathon” held in Berlin, Germany. This study aimed to examine trends in participation and performance in the “Berlin Marathon” on all its previous 46 editions from 1974 to 2019, the largest dataset ever studied in this event with 696,225 finishers (after data cleaning). Athletes in all age groups increased their participation, except for male athletes aged 20–49 years and athletes of both sexes above 79 years of age. This overall increase in participation was more pronounced in women, but still, there are more men than women participating in “Berlin Marathon” nowadays. All age group athletes decreased their performance across years overall, whereas the top ten recreational athletes improved their performance over the years. Our findings improved the knowledge about the evolution of male and female marathoners across calendar years, especially for the fastest marathon race in the world, the “Berlin Marathon.”

## Introduction

The inaugural modern marathon event was first held during the 1896 Summer Olympic games and was refined to the official distance of 42.195 km for the 1908 Olympic Games in London (Burfoot, 2007; Wilcock, 2008). However, these races were only for men. The women’s marathon event was added to the official program nearly 99 years later, during the 1984 Los Angeles Olympic Games (Burfoot, 2007). In the 1970s and 80s – in line with the upcoming fitness trend – a skyrocketing boom of these marathon events occurred (Valentine, 1982; Maffetone et al., 2017; Knechtle et al., 2018) and marathon races have become more and more popular all over the world (Vitti et al., 2020). The largest participation numbers so far were reached in 2016, with approximately nine million runners crossing finish lines all over the world (The State of Running, 2019). A total of 12% of those finishers were marathoners (The State of Running, 2019), and most of them were age group athletes (Rüst et al., 2013; Lara et al., 2014).

With such participation numbers and demographic diversity, marathon running provides scientifically interesting samples for research in endurance sport (Stones and Baker, 2020). Further analyses of these data can contribute to, e.g., a better understanding of aging processes and consecutively age-related performance declines (Leyk et al., 2007; Reaburn and Dascombe, 2008; Tayrose et al., 2015), differences between the sexes regarding physiology and performance (Rüst et al., 2013; Knechtle and Nikolaidis, 2018; Nikolaidis et al., 2018, 2019c), the influence of lifestyle (Leyk et al., 2009, 2010), environmental (Nielsen, 1996; El Helou et al., 2012; Maffetone et al., 2017; Knechtle et al., 2019) and demographic factors (Knechtle et al., 2017; Maffetone et al., 2017; Nikolaidis et al., 2017; Knechtle and Nikolaidis, 2018) on performance and a better understanding of motivational factors for running of different cohorts and decades (Krouse et al., 2011; Nikolaidis et al., 2019b).

The “Berlin-Marathon,” for the first time in 2019, marked the final race of the “World Marathon Majors,” a series of six of the largest and most renowned marathons in the world and popularly known as the “Marathon’s Champion’s League” (World Marathon Majors, 2021). T his may be a deserved position as today, the “Berlin Marathon” ranks third in the world regarding the size of the runner field and is the fastest course among the city marathons worldwide in men’s racing and third fastest in women’s (Berlin Marathon, 2021). Seven of the ten fastest men’s marathon times and numerous world records were set during the “Berlin-Marathon” (Berlin Marathon, 2021). Among those records is the current men’s world record, achieved in 2018 by the Kenyan Eliud Kipchoge in a time of 2:01:39 (World Athletic Records, 2021). The current course record for women in “Berlin-Marathon” was set by Kipchoge’s countrywomen Gladys Cherono in 2018 (2:18:11 h) (BMW Berlin Marathon, 0000). This running time is close to the current world record for ‘‘women only marathon’’ (as is also ‘‘Berlin Marathon’’ since 2011) set by Mary Jepkosgei in 2017 in the ‘‘London Marathon’’ with a time of 2:17:01^1^.

More recently, research has tended to focus primarily on marathon races held in the United States, with the “New York City Marathon, 2021” – the largest marathon race nowadays – being one of the most investigated (Jokl et al., 2004; Santos-Lozano et al., 2015; Zavorsky et al., 2017; Nikolaidis et al., 2018). Alongside the “New York City Marathon, 2021” is the “Boston Marathon, 2021” generating the next most scientific interest (Maffetone et al., 2017; Knechtle et al., 2018, 2020). Most studies conducted on those two marathon events (the “New York City Marathon, 2021” and the “Boston Marathon, 2021”) have demonstrated increasing participation rates over the last two decades (more pronounced in women than in men) and a concomitant increase in mean race times (i.e., decreased performance) across calendar years (Jokl et al., 2004; Mathews et al., 2012; Nikolaidis et al., 2018; Knechtle et al., 2020). Even though the “Berlin-Marathon” is one of the fastest and most popular marathon events in the world, there has not been any complete analysis of participation and performance trends on all its 46 previous editions. Therefore, it is interesting to see whether the above-mentioned consensus about the development of participation rates and running times found in American marathon events would also be applicable for European Marathon races such as the “Berlin Marathon.”

Much of the current research on participation and performance in marathon racing also was done with regard only at a short period of time or limited participation group. For example, Ahmadyar et al. (2015, 2016) studied elderly marathoners (>75 years of age) in the four largest marathon events nowadays in the time period from 1990 to 2014 and 2004 to 2011. Knechtle et al. (2018) analyzed participation and performance for all editions of “Boston Marathon” from 1879 to 2017, but only in male runners. Jokl et al. (2004) included runners of all age groups and both sexes in their analysis of the “New York City Marathon,” but only analyzed the time period between 1983 and 1999 (Jokl et al., 2004). Mathews et al. (2012) studied the mortality among marathon runners in the United States, and thereby analyzed a variety of events throughout the United States but could only include data from 2000 to 2009 in their study. More recently, an interesting perspective that investigated the motivation for running in the “Athens Classic Marathon” was done by Nikolaidis et al. but only focused on the 2017 edition (Nikolaidis et al., 2019b). Therefore, by analyzing the full data of the “Berlin Marathon” since its inaugural event in 1974, we hope to provide valuable new information to the above-mentioned ongoing research.

Furthermore, the current study aims to contribute to the growing literature on women’s participation in endurance sports. Previously, women were barred from participating in sporting events primarily based on Victorian area myths about endurance exercise and the fragility of the female body (Wrynn, 2014; Hill, 2017). It has taken several decades for women to be permitted the same sporting opportunities as their male counterparts, and even still, equity has not been achieved (Capranica et al., 2013). The first time women were permitted to officially run a marathon race occurred during the 1972 “Boston Marathon” (Thibault et al., 2010; Knechtle et al., 2020). In 2018, for the first time in history, equal participation between men and women in running events was achieved with women representing 50.24% of runners at events all around the world (The State of Running, 2019). Race organizers at the “Berlin-Marathon” in its 46th edition (2019) set its focus for the first time officially on the women’s race (Berlin Marathon, 2021).

Although progress toward equal opportunity has been consistent for female athletes, sports science research focusing on women is still sorely lacking. Only 4% of the present research in sport sciences is conducted exclusively on female athletes, whereas 27% of those studies are conducted exclusively on male athletes (Women in Sports are Often Underrepresented in Science, 2016; Costello et al., 2014). Further, an analysis of 1,382 articles published from 2011 to 2013 showed that female participation rate per article was around 36 percent (added up in this analysis was a total of more than six million participants) (Women in Sports are Often Underrepresented in Science, 2016). As physiology and biomechanics properties differ between the sexes, it is not applicable to transfer study results found in a predominantly male population to a female population, which – as mentioned – accounts for more than half of the athletic population currently competing in running (Women in Sports are Often Underrepresented in Science, 2016; Lewis et al., 1986). To optimize female performances and health in sport, we need to include women in our analyses in order to better understand the peculiarities that may exist in physiology. Therefore, we are happy to enrich the existing pool of knowledge with more data on female participation and performance in marathon racing.

Taken together, our understanding of the characteristics of participation and performance are well known for only a handful of important marathon events (Jokl et al., 2004; Mathews et al., 2012; Santos-Lozano et al., 2015; Maffetone et al., 2017; Zavorsky et al., 2017; Nikolaidis et al., 2018, 2019c; Knechtle et al., 2020) and/or limited periods or participation groups (Jokl et al., 2004; Mathews et al., 2012; Ahmadyar et al., 2015, 2016; Nikolaidis et al., 2019b). Drawing general conclusions out of these limited data, which can be important for the above-mentioned research fields in sport, epidemiological and medical sciences, should be done cautiously.

The aims of the present study were, therefore, (i) to analyze the changes in participation and performance trends of age group marathon runners in the “Berlin-Marathon” for all its previous editions, (ii) to compare the sex differences in performance as a function of age across the years, and (iii) by this to provide one more complete analysis on participation and performance of female athletes in the history of a significant event in order to allow best possible future findings of particularities in female sports physiology. Based upon existing evidence, we hypothesized that for “Berlin-Marathon” between 1974 and 2019, the participation of all age groups would grow, with more substantial growth in female participation and, therefore, a narrowing sex gap in participation. Further, we hypothesized the performance of top age group athletes would improve over calendar years, whereas the performance of the average age groupers would decrease.

## Materials and Methods

### Ethics Approval

The institutional review board of St Gallen, Switzerland, approved this study (EKSG 01/06/2010). Since the study involved the analysis of publicly available data, the requirement for informed consent was waived.

### Participants

To test our hypothesis, data (i.e., first and last name, sex, age, calendar year, and running time) on all successful female and male finishers in the “Berlin-Marathon” since 1974, the inauguration year of the “Berliner Volksmarathon,” was obtained from the official race website (BMW Berlin Marathon, 0000). To compete in the “Berlin-Marathon,” athletes must be 18 years old or older but must not meet specific time standards (BMW Berlin Marathon, 0000). Starting places are limited and assigned via raffle. Initially, 884,927 finishers were considered in our analysis.

### The Race

The “Berlin-Marathon” takes place from mid to end of September, depending on several logistical factors. The course builds one large loop through the historic city of Berlin, with the finish line lying almost under the “Brandenburger Tor.” Berlin lies 34 meters above sea-level, the average temperature in September is about 14.9°Celsius and average humidity about 75% (Klimatabelle, 2021) and the total elevation of the course is only 50 meters (Berlin Marathon, 2021).

### Data Analysis

First and last name, sex, age, calendar year, and running time on all successful female and male finishers in the “Berlin-Marathon” from 1974 to 2019 were obtained from the official race website (BMW Berlin Marathon, 0000). We cleaned the dataset removing runners with missing or questionable (unreliable) information on race time, i.e., race time under 2 h or over 6 h. With respect to age stratification, finishers were classified in 10-year age groups (e.g., 20–29 years to 70–79 years) to analyze performance and participation. We compared top ten age group runners to the age group average in order to highlight differences in performance over calendar years between those groups and both sexes. Further, we compared top five age group runners of each age-category to average age group runners of the same age category to examine performance declines during aging.

### Statistical Analysis

All statistical procedures were carried out using the Statistical Package for the Social Sciences (SPSS version 26. IMB, IL, United States) and GraphPad Prism (version 8.4.2. GraphPad Software LLC, CA, United States). The Shapiro-Wilk and Levene’s tests were applied for normality and homogeneity, respectively. Six General Linear Models (two-way ANOVA) were used as follows: model 1 – all participants by sex and calendar year; model 2 – top ten athletes in each race by sex and calendar year; model 3 – all participants by age group and sex; model 4 – top five athletes in each age group in each race by age group and sex; model 5 – all men participants by age group and calendar year; model 6 – all women participants by age group and calendar year. When interactions were found (*p* < 0.05), pairwise comparisons were applied to identify the differences more accurately. Calendar years without sex or age information were removed from the analysis. For performance analysis, age groups, or calendar years with less than five participants were removed. Applying those criteria, the calendar years 1976 to 1984, 1994 to 1998, and 2019 had to be removed from the performance × years for men and women analysis, the calendar years 1976 to 1978, 1980 to 1981, 1994 to 1998 and 2019 had to be removed from the performance × years × age group analysis for men and the calendar years 1974 to 1984, 1994 to 1998 and 2019 had to be removed from the performance × years × age group analysis for women. For the participation × year analysis for both sexes (total participants) we had to remove the years 1976 to 1984, 1994 to 1998 and 2019, for the participation × sex × age analysis, we had to remove the years 1974, 1976 to 1978, 1980 to 1981, 1994 to 1998 and 2019 for men and the years 1974, 1976 to 1984, 1994 to 1998 and 2019 for women. The level of significance utilized was *p* ≤ 0.05.

## Results

**FIGURE 1:**
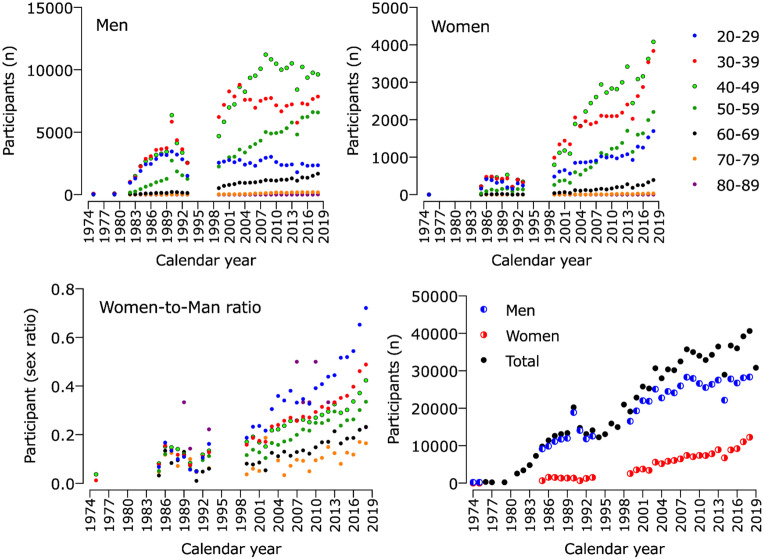
Number of participants in “Berlin Marathon” from 1974 to 2019 by sex and age groups.

The total number of athletes ever registered in the 46 editions of the “Berlin-Marathon” between 1974 and 2019 was 884,927 finishers. After filtering out invalid data regarding our criteria, 696,225 finishers were included for the final analysis. The number of athletes participating in the “Berlin-Marathon” increased from only 236 men and 8 women in 1974 to 28,373 men and 12,268 women in 2018 (Figure 1). Moreover, all age groups increased their participation, except for male athletes aged 20–49 years and athletes of both sexes above 79 years old (Figure 1).

**FIGURE 2:**
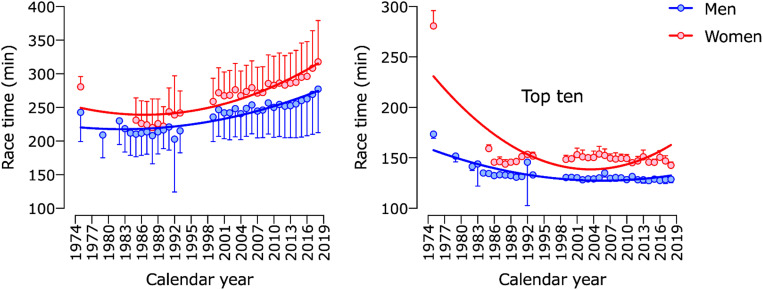
Race time of men and women in “Berlin Marathon” across calendar years.

The first two ANOVA models (model 1 and 2) to analyze performance showed a significant effect on sex, year, and interaction sex × year (Table 1). However, pairwise comparisons and the linear trend show that model 1 tends to increase race time across calendar years. In contrast, in model 2, which only included the top ten athletes in each race, race time has a decreasing trend across calendar years (Figure 2).

**TABLE 1:** ANOVA results of performance analysis in “Berlin marathon” in different models.

	**Factor**	***F***	***p*-value**
Model 1	Sex	294.5	<0.001
	Year	29.0	<0.001
	Sex × Calendar year	85.4	<0.001
Model 2 (Top ten athletes)	Sex	91.6	<0.001
	Year	3.4	0.001
	Sex × Calendar year	12.8	<0.001
Model 3	Sex	280.4	<0.001
	Age group	155.3	<0.001
	Sex × Age group	19.9	<0.001
Model 4 (Top five athletes in each age group)	Sex	29.5	0.001
	Age group	38.7	<0.001
	Sex × Age group	13.1	<0.001
Model 5 (Men)	Age group	208.3	<0.001
	Calendar year	80.0	<0.001
	Age group × Calendar year	7.6	<0.001
Model 6 (Women)	Age group	100.4	<0.001
	Calendar year	39.2	<0.001
	Age group × Calendar year	1.8	<0.001

**FIGURE 3:**
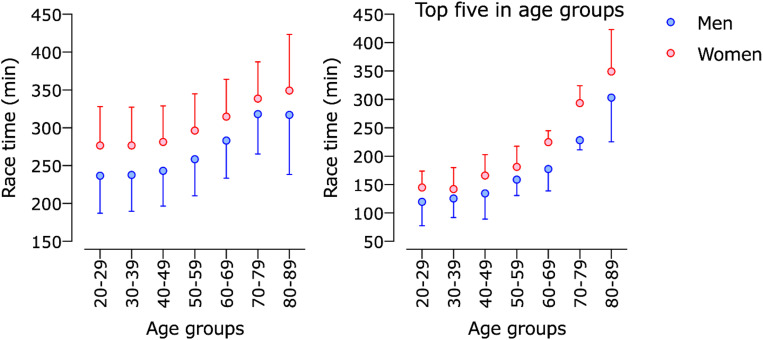
Race time of men and women in “Berlin Marathon” across age groups.

Models 3 and 4 showed significant effects on sex, age group, and interaction sex × age group (Table 1). Both models showed similar performance trends across age groups, with the lowest race times between 20 and 39 years old, and slight increases across each next age group (Figure 3).

**FIGURE 4:**
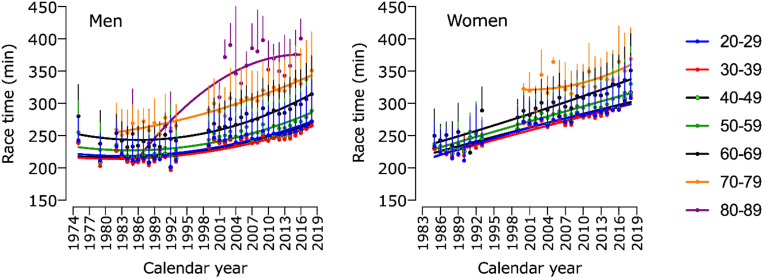
Race time of men and women in “Berlin Marathon” in age groups across calendar years.

Models 5 and 6 showed significant effects for age group, year, and an interaction age group × year (Table 1). Both men and women showed similar performance trends among all age groups, with increasing race time across calendar years (Figure 4).

## Discussion

The main aims of this study were (i) to analyze the changes in participation and performance trends of age group marathon runners in the “Berlin-Marathon” for all its previous editions, (ii) to compare the sex differences in performance as a function of age across the years, and (iii) by this to provide one more complete analysis on participation and performance of female athletes in the history of a significant event in order to allow best possible future findings of particularities in female sports physiology. The main findings were that (i) the number of finishers increased for both women and men runners over the decades, (ii) this increase in participation was more pronounced in women than in men, and (iii) all age group athletes decreased their overall performance, whereas the top ten recreational athletes improved their performance over the years.

### Participation Trends

The first important finding was that the number of finishers increased for both women and men runners over the decades, which supports our first hypothesis. In the first edition of the “Berliner Volksmarathon” in 1974, only 236 men and 8 women took part, and by 2018 there were 28,373 men and 12,268 women participating. The increase in participation in recent years was more pronounced in women, whereas men of the age groups 20 – 49 years showed a slight decrease in participation numbers in the last two decades. This narrowing of the gender gap in participation has been previously reported in well-studied city marathons such as the “New York City Marathon” (Hunter and Stevens, 2013; Nikolaidis et al., 2018) and the “Boston Marathon” (Knechtle et al., 2020). Some researchers even predicted a closing of the gender gap in participation in the marathon; however, it has n*o*t appeared yet for the large city marathons such as the “New York City Marathon” (Jokl et al., 2004; Knechtle et al., 2020; Vitti et al., 2020) and after analysis of the present study, neither in the “Berlin-Marathon” so far.

Still, an extensive but not officially validated research of Andersen et al. showed that in 2018 – “for the first time in history” – there were more female than male runners competing worldwide (50.24% women) (The State of Running, 2019). The analysis by Andersen and colleagues covered 96% of US-based running race results, 91% of the race results from the EU, Canada, and Australia and a “big portion” from Asian, Africa, and South America. Andersen et al. analyzed not only marathon races, but all types of running events. This may be the crucial point: Women seemed to be more engaged in shorter running events. Moreover, it was noted that the longer events were, women were less likely to participate showing a decreased number of starters in relation to men. However, further investigation in worldwide participation in running events of different distances considering demographical conditions of the participants is required. However, also an analysis of running races from one single country would be of interest.

A significant observation described previously by Nikolaidis et al. (2018) found that women, in general, start and stop racing at a younger age than men. That men-to-women-ratio in the older age groups compared to their younger counterparts could be explained through the existence of historical and social barriers (Vitti et al., 2020). Pointing in the same direction are the findings of Andersen et al., who – by comparing sex participation rates for running events between different countries – found a clear correlation between “general gender equality” and equality in participation rates (The State of Running, 2019). When taking into account the historical perspective of women’s competitive sports, female athletes have been subjected to a variety of discriminatory practices and gender-based social barriers, many of which are still ongoing (Costello et al., 2014). The 2012 Summer Olympic Games were an important milestone, whereby every participating country’s delegation included at least one female competitor (Costello et al., 2014), albeit a positive step, but far from what is required for a true closing of the gender gap.

However, it must be noted that some countries have been successful in improving women’s participation rates and some have even tipped the scales completely. Higher participation rates in running events for women than for men have been observed with Iceland on the top of the board (59%), followed by the United States (58%) and Canada (57%) (The State of Running, 2019). Switzerland (16%) and Italy (19%) are among the countries with the least female participation in running events (The State of Running, 2019). In the case of Switzerland, this participation rate seems to contradict to the fact that 49% of the regularly running population in Switzerland (which is about one-third of the Swiss population) are women (Sport Schweiz 2020, 2020). This finding may be quite surprising, given the mentioned country’s apparent progressiveness, but not so, if one considers the repeated international criticism on Switzerland’s gender policy (Human Rights Switzerland, 2021). When examining sports such as road cycling, the gender gap in Switzerland seems even more prominent (in 2019, only 14% of the licensed road cyclists in Switzerland were women) (*personal and unpublished written communication with Stefania Ratano, the responsible for members and licenses at the Swiss national cycling federation “Swiss Cycling,” mid of June 2020*). This possible correlation between actual and effective “general gender equality” and participation rates in endurance events could be an interesting subject for future research.

### Performance Trends

As expected, performance in all age group athletes decreased, whereas the top ten age group athletes improved their finishing times across calendar years. This tendency was found for the “Boston Marathon” already (Maffetone et al., 2017; Knechtle et al., 2019, 2020). Vitti et al. (2020) analyzed 1.2 Million runs “during half a century” in the “New York City Marathon” and described the phenomenon of “the faster get faster and the slower get slower.” They stated that nowadays, more women, more recreational and more elderly runners participate in most of the marathons worldwide, while in the 1970s, participation was limited to mainly elite male runners (Vitti et al., 2020). Knechtle et al. (2018) who analyzed men’s participation and performance in the Boston Marathon from 1897 to 2017 commentated the same way: There is more variability on performance introduced by the increased number of age group runners in marathon running. We also see these changes in the marathoners’ community over the years as an important factor that explains why “the slower get slower.”

The factors that influence the improvement in running times of the already fast marathoners seem to be more complex and multi-factorial. Historically, marathon running training, pre-race preparation, nutrition, fluids and equipment were significantly different than what is available today (Ineos 1:59 Running Challenge, 2019; Joyner et al., 2020). Special attention should be given to recent advancements in running shoe technology and as a result, improved running times by professional runners, who primarily wear them (Carbon Fiber Racing Shoe Battle, 2020). In 2017 “Nike” released the first carbon fiber shoe, triggering a technology advancement race between commercial shoe companies (Carbon Fiber Racing Shoe Battle, 2020). Independent tests showed significantly lower oxygen uptake by runners at higher running speeds wearing carbon sole “Nike” shoes (Carbon Fiber Racing Shoe Battle, 2020; Hoogkamer et al., 2018; Hunter et al., 2019).

Therefore, improvements in “running economy” seem to be crucial for the constantly dropping running times. This is also seen in analyses of East-African runners, who comprise most of today’s elite in big-city-marathons (World Athletic Records, 2021). Those runners, who originate from specific regions in Kenya and Ethiopia (Scott et al., 2003; Onywera, 2009), show a profile that allows them to run with an exceptional high running economy (Weston et al., 2000; Lucia et al., 2006; Kong and De Heer, 2008; Mooses and Hackney, 2017). This outstanding running economy is seen as one of the important factors for the dominance of East-African runners (Weston et al., 2000; Kong and De Heer, 2008; Mooses and Hackney, 2017). Still, even if the reasons for Kenyan and Ethiopian apparent dominance in endurance running races have been deeply analyzed, there remains no clear consensus on what contributes to their dominance (Hamilton, 2000). So, one part of the explanation why the “faster” become faster, could be a growing participation rate of mentioned African ethnicities in the big city marathons during the last decades (Vitti et al., 2020). Still, extensive research about worldwide participation rates in marathon running for the last decades split up in ethnicities or countries of origins are missing.

Not all studies conducted on a large population of endurance athletes find improving race times of the “fast athletes” and slowing race times of recreational athletes in the past though. Research conducted on elite and master Ironman triathletes e.g., showed improving race times for both these groups of athletes during the last decades while also the average age of the athletes augmented (Lepers et al., 2013; Gallmann et al., 2014). Regarding at these results, the importance of interaction of age of a certain study population and age of peak performance in endurance sport has to be underlined. Future studies need to take into consideration the current ideas about age of peak performance (see below) in a certain discipline and changing age of participations over decades of analysis.

Athletes in the age groups 20–29 years and 30–39 years showed the fastest race times in the “Berlin-Marathon” for both sexes, as well as the top five were the fastest in those two age groups. For the following older age groups, we constated slight increases in race times. Above the age of 60 years, the increase in average race time was more pronounced, with a greater decline of performance for top five age group athletes than for the average age group. All those findings account for both sexes, with only minor differences in-between.

There is no consensus about the precise age of peak performance and the dynamics of the age-related performance decline in endurance sport in the current scientific literature (Lara et al., 2014; Zavorsky et al., 2017; Nikolaidis et al., 2018, 2019a; Jäckel et al., 2020). Depending on the discipline (“locomotion models”) (Jäckel et al., 2020), the study population (recreational athletes versus top age group athletes (Lepers and Cattagni, 2012; Zavorsky et al., 2017) versus top professional athletes (Knechtle et al., 2018, 2019) and other factors like research period (Leyk et al., 2007; Lara et al., 2014; Knechtle et al., 2018), the outcomes are different. For example, Jäckel et al. (2020) stated a progressive running performance decline for recreational half-ironman triathletes after the age of 50 years. The same was constated for age group marathoners by Zavorsky et al. (2017) who examined data from the New York City, Boston and Chicago marathons, in addition to Leyk et al. (2007) who examined 69 marathons and 65 half-marathons performed between 2003 and 2005 in Germany. At the same time, Käch et al. (2018), who investigated recreational Ironman triathletes, reported a much earlier decline in running performance starting at about 30–34 years in women and 35–39 years in men. Reaburn et al. summarized the same for recreational runners (Reaburn and Dascombe, 2008). Their review reported that the declines in performance are curvilinear from age 35 years until approximately the age of 60–70 years (Reaburn and Dascombe, 2008). Additionally, Lepers et al. (2010) showed that elite triathletes maintained their performance up to the fourth or fifth decade of life, i.e., a curvilinear decline from 50 years onward in Olympic Triathlon World Championships and from 45 years onward in the “Ironman Hawaii.”

Also, there is the question of sex and age of peak performance, which is important for athletes and coaches to plan a career (Allen and Hopkins, 2015). In contrast to existing findings reporting a higher age of peak marathon performance in women compared to men (Nikolaidis et al., 2018), it was found that women achieved their best marathon race time ∼5 years earlier in life than men by analyzing all finishers of the “New York City Marathon” between 2006 and 2016 (Nikolaidis et al., 2018). More data from big events over large periods of time is needed to discuss those questions and find consensus about the age of peak performance and the dynamics of the age-related performance decline in endurance sport.

### Limitations

Several limitations of this study should be noted. First, the data obtained for the “Berlin Marathon” database only included finishing times, gender and age of the participants. Other factors, such as training volume and intensity, previous experience, ethnicity and physiological variables (VO_2_max, lactate threshold, and running economy) were not recorded. Still, those other factors are known to affect endurance running performance, and therefore some part of the variance in endurance performance explained by age may actually be related to those (Lara et al., 2014). For this reason, the outcomes found in this investigation should be reinforced by collecting experimental data. Second, there are limited participation places for the ‘‘Berlin Marathon,’’ which are allocated by raffle. Information about the year of installment of the raffle and places distributed per year and sex since then are not available^2^. If there had been a distribution key respecting the sex of participants, it consecutively must have had an influence on sex ratios found in finisher reports and thereby on our results. At the same time, drawing lots shouldn’t influence mean running times because the sample sizes in “Berlin Marathon” are large enough to ensure an evenly distributed composition of the runners. In the present study, we only considered finishing numbers but neither registration nor starting numbers, which are not accessible to public “(see text footnote 2)”. By this, we cannot exclude to actually report more on the ability of participants to make it to the finish line within the limit of 6 h, than on the actual trend to participate and register for the “Berlin Marathon.” We do not expect the latter to differ by much, still those analyses would be needed to confirm our findings. Third, sex and age data are unavailable for the years 1976 to 1984, 1994 to 1998 and 2019. For those years, the analyses are missing between sexes, age groups, and sex ratio. Therefore, our analysis on that subject is not fully complete. Nevertheless, the available data allows us to see trends and make statistically reliable statements on sex ratios. Further, the way the platform of “Berlin Marathon” displays the data about participants has changed throughout the years, so as how they store data (BMW Berlin Marathon, 0000). For some years, we suspect that they calculated the age of each participant as “current year – year of birth.” This can generate a problem with participants with missing data because they would enter as 90 + years old, and we excluded this age group from our analysis. Finally, in order to make statements about age of peak performance and the dynamics of age-related performance declines in marathon running, data should be split up into rather small age group fragments. The present work analyzed age groups in 10-year intervals, which doesn’t allow us to draw conclusions regarding this issue.

## Conclusion

This study tested the hypothesis that for “Berlin-Marathon” over all its previous 46 editions (1974–2019), participation of age group athletes would increase, the over-all performance of age group athletes would decrease and top age-group performances would improve over calendar years. Participation for female and male runners increased, with a stronger increase in female participation and thereby narrowing sex gap in participation, the fastest age group women and men became faster across years and average age group performance decreased. This is the largest dataset ever studied in this event and provides valuable information in the ongoing research about characteristics in participation and performance in large city marathons. Future studies might investigate the influence of other parameters such as country of origin, training volume, training years and motivation of athletes to understand how training and life of athletes can be planned best to achieve maximal performance.

## Data Availability Statement

The raw data supporting the conclusions of this article will be made available by the authors, without undue reservation.

## Author Contributions

MR and BK designed the study. EV collected the data. CS performed the statistical analyses. JA, LH, TR, and PN contributed by writing and editing a part of the manuscript. All authors read and approved the final manuscript.

## Conflict of Interest

The authors declare that the research was conducted in the absence of any commercial or financial relationships that could be construed as a potential conflict of interest.

## Footnotes

1 www.worldathletics.org/records/by-discipline/road-running/marathon/outdoor/women

2 www.bmw-berlin-marathon.com/en/your-registration/registration-information/general/
